# Leveraging sequential least-cost modelling to uncover multiple introductions: a case study of an invasive wild bee species

**DOI:** 10.1007/s10980-025-02188-9

**Published:** 2025-08-14

**Authors:** Christa Rohrbach, Gudrun Wallentin, Jovana Bila Dubaić, Julia Lanner

**Affiliations:** 1 Department of Geoinformatics – Z_GIS, Paris-Lodron-University of Salzburg, Schillerstr. 30, 5020 Salzburg, Austria; 2 Faculty of Biology, University of Belgrade, Studentski Trg 16, Belgrade, Serbia; 3 Department of Environment and Biodiversity, Paris-Lodron-University of Salzburg, Hellbrunner Str. 34, 5020 Salzburg, Austria

**Keywords:** Dispersal, Expansion rate, Invasive species, Least-cost modelling, *Megachile sculpturalis*, Multiple introductions

## Abstract

**Context:**

Invasive species pose a significant threat to biodiversity, creating a need for accurate methods to assess their spread. Although multiple introductions are common, estimates of expansion rates often assume a single introduction site due to limited knowledge of population structure.

**Objectives:**

This multidisciplinary study aimed to develop a novel spatio-temporal approach to delineate potential populations without prior knowledge of population structure. We applied this approach to the Sculptured Resin Bee, Europe’s first non-native bee species, providing regional expansion rate estimates for its spread across Europe.

**Methods:**

Observation data from 2008 to 2024 were analysed. Based on an environmental suitability map, sequential least-cost modelling was applied in annual time steps, linking each new observation to the nearest known observation via a least-cost path. Populations were delineated by excluding high-cost paths and analysing the connectivity of the remaining paths, and expansion rates were calculated using the distance regression method.

**Results:**

We identified two populations, which align with known genetic groups in the area of France, Switzerland and Austria. Our modelling results also indicate two additional populations introduced to Italy and Serbia. Expansion rates ranged from 13.3 km/year to 58.6 km/year and peaked at 89.7 km/year during expansion phases, exhibiting a consistent sigmoidal expansion pattern.

**Conclusions:**

Our spatio-temporal approach delineates introduced populations without prior genetic knowledge, improving expansion rate estimation and informing targeted genetic sampling, monitoring, and management efforts of invasive species.

**Supplementary Information:**

The online version contains supplementary material available at 10.1007/s10980-025-02188-9.

## Introduction

Invasive species are organisms transported outside their natural range by human activities, either intentionally or accidentally, thereby overcoming biogeographic barriers (Richardson et al. 2011). Although definitions vary, invasive species are typically characterised by their ability to establish, spread, and cause environmental or socio-economic harm (IUCN 2019). Invasive species are a major driver of biodiversity loss (Bellard et al. 2016). Accurately estimating their spread is crucial for developing management strategies as for example the designation of suitable zones for surveys, quarantine and control measures (Tobin et al. 2007).

The invasion process is often observed in distinct temporal phases, including an initial lag phase of minimal population growth and spatial expansion (Crooks 2005; Hui and Richardson 2017). Strong dispersal abilities, such as those of flying insects, are often associated with sigmoidal expansion patterns, where the lag phase is followed by a rapid expansion and a slow final phase (Hui and Richardson 2017). To account for different phases with different dynamics of spread (cf. Arim et al. 2006; Hui and Richardson 2017), expansion rates need to be calculated separately for each phase. Sandvik (2020) argues that the *highest realistic* expansion rate that is estimated for temporal phases should be used in invasive species management to assess the species’ dispersal potential.

Expansion rates can be estimated using various methods, such as distance regression, square root area regression, and boundary displacement methods (Liebhold et al. 1992; Gilbert and Liebhold 2010; Hui and Richardson 2017). In a performance comparison against a simulated expansion with a known expansion rate, Gilbert and Liebhold (2010) identified distance regression as the most robust method to small samples and sample point distribution as compared to square root area regression and boundary displacement. However, all of these methods are typically applied to the entire observation data in a study area. By doing so, it is assumed that the spread originates from a single population, overlooking the frequent occurrence of multiple introductions (Bossdorf et al. 2005; Ellstrand and Schierenbeck 2006; Lawson Handley et al. 2011), as evidenced by genetic studies across various animal taxa including reptiles (Kolbe et al. 2004), gastropods (Facon et al. 2008), mammals (Zalewski et al. 2010), insects (Miller et al. 2005; Ortego et al. 2021; Lanner et al. 2021), and crustaceans (Robinson et al. 2018). The presence of distinct populations is sometimes accounted for by manually dividing observation records along spatial barriers and calculating expansion rates for each population separately (Tobin et al. 2007; Fraser et al. 2015), but this requires prior knowledge of population structure, for example obtained through labor-intensive genetic methods, which are often not available, especially not at an early invasion stage.

Considering the challenges associated with dividing observation records into populations, landscape connectivity modelling methods offer a promising approach to infer population structure from dispersal pathways without prior genetic information. Several methodological frameworks exist to model dispersal based on landscape connectivity, including approaches based on (1) Agent-based modelling, (2) Cellular Automata, (3) Circuit Theory, and (4) Least-Cost modelling, each with distinct theoretical foundations and practical implications.

Agent-based modelling (1) has been used to simulate expansion of populations (Bocedi et al. 2014; Lustig et al. 2019), based on a well-known behaviour of individuals. Its focus is on conceptual understanding of emergent phenomena like range expansions rather than on data-driven analysis. Cellular Automata (2) are closely related to Agent-based models with the difference that they represent dispersal as a local spread process between adjacent cells. Both methods simulate dispersal mechanistically, which makes them well suited for testing management and pest control scenarios, but they are conceptually not well suited for dividing given observation data into populations.

Circuit Theory (3), a theoretical framework from electrical engineering describing conductance principles, was adapted to landscape connectivity modelling by McRae et al. (2008) and has since been rapidly adopted across ecological disciplines (Dickson et al. 2019). While powerful for predicting gene flow due to simultaneously accounting for multiple pathways (Dickson et al. 2019), it generates current density maps rather than discrete paths and thus requires post-processing to obtain such.

Least-cost modelling (4) has been widely used in ecology to identify corridors between habitat patches and quantify habitat connectivity (Zeller et al. 2012; Etherington 2016). Originally developed in the field of transport geography (Turner and Miles 1971), it gained traction in ecological disciplines in the late 1990s (Adriaensen et al. 2003). Both least-cost modelling and Circuit Theory based approaches rely on representing landscapes as raster surfaces, where movement is impeded to varying degrees, commonly referred to as cost surfaces or resistance surfaces. In its original implementation, least-cost modelling involves converting this cost surface to a graph, followed by the application of a shortest-path algorithm to identify an optimal path (least-cost path) between points of interest. It is typically used in a static context, even when applied to analyse spread dynamics (Mineur et al. 2010; Kaláb et al. 2021).

Dynamic processes, such as the spread of invasive species, can be best reconstructed when explicitly incorporating the temporal dimension in the model. Sequential least-cost modelling addresses this need by iteratively applying the method over time steps, allowing changes in the input data to be considered in each step. It has been used in archaeology to simulate ancient seafaring, enabling a virtual navigator to adapt to changing wind conditions in time steps (Perttola 2022). The incorporation of the temporal dimension makes sequential least-cost modelling particularly suitable for analysing the spatio-temporal dynamics of biological invasions.

The aim of this study is to propose a sequential least-cost modelling based approach as a data-driven, spatio-temporal way to delineate distinct populations of an invasive species and to derive their respective expansion rates. The Sculptured Resin Bee (*Megachile sculpturalis*), the first invasive bee to spread across Europe (IUCN 2019), serves as a model for a multiply introduced invasive species. Genetic data suggests at least two independent introductions in the area of France, Switzerland, and Austria (Lanner et al. 2021), making this bee an ideal case study. Using comprehensive observation data and a habitat suitability map (Lanner et al. 2022), we (1) apply sequential least-cost modelling to connect each observation to the nearest previous observation via a least-cost path in annual time steps, establishing the foundation to (2) delineate populations by excluding high-cost paths and analysing the connectivity of the remaining paths, and (3) provide the first expansion rate estimates for the Sculptured Resin Bee outside its native range by applying the distance regression method to the delineated populations.

## Methods

**Fig. 1:**
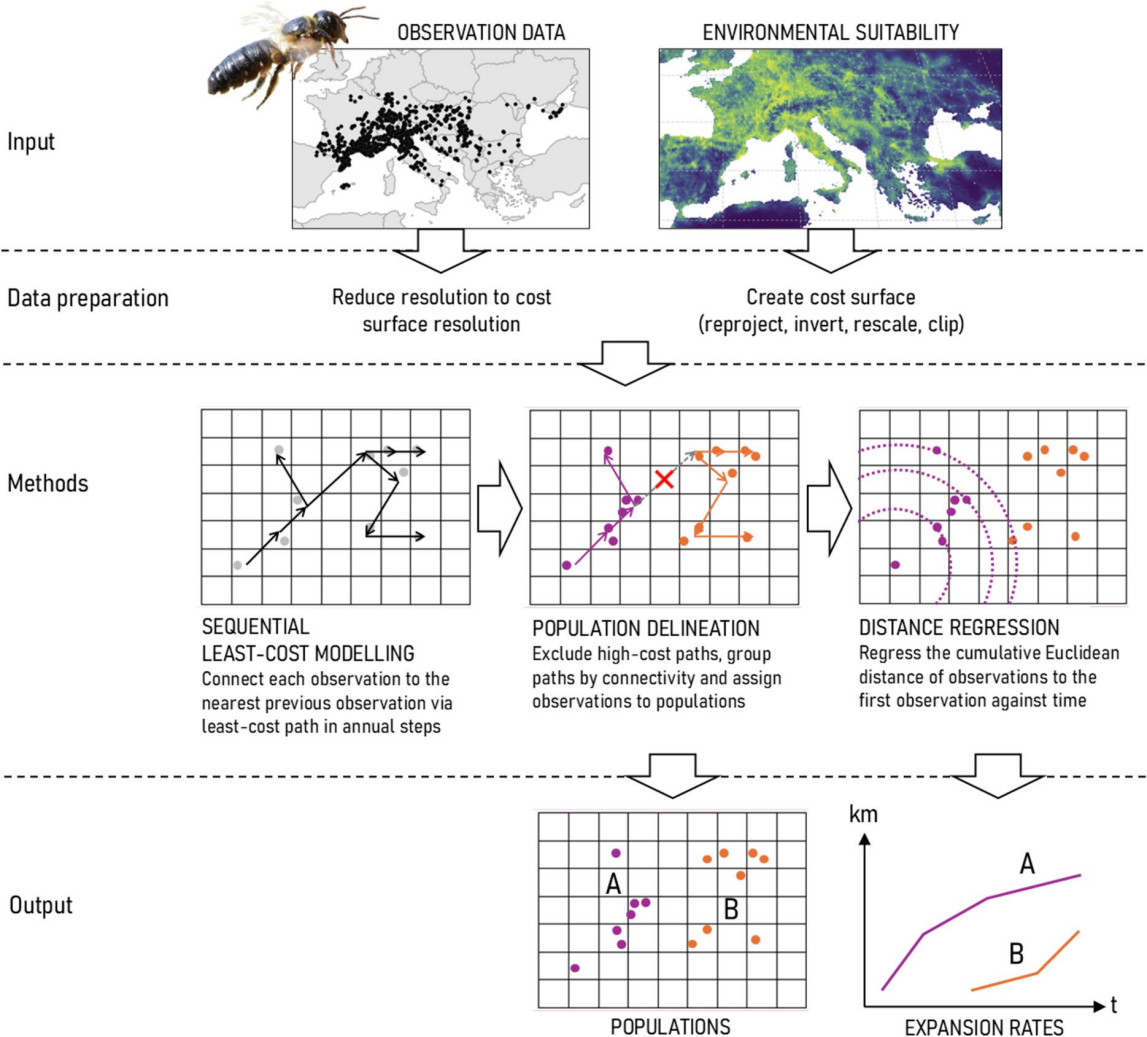
Using observation data of the Sculptured Resin Bee and an environmental suitability map (Lanner et al. 2022), we proceeded in the following steps: (1) sequential least-cost modelling to connect each observation to the nearest previous observation via least-cost path in annual time steps, (2) population delineation by excluding high-cost paths and analysing the connectivity of the remaining paths, and (3) calculation of expansion rates for the delineated populations using the distance regression method

The methodological framework presented here provides an approach for analysing the spatio-temporal expansion dynamics of invasive species. The core framework, excluding the cost surface creation, is implemented as standalone Python scripts, made available at https://github.com/thunwal/bioinvasionanalysis/releases/tag/v1.1.0. As illustrated in Fig. 1, the workflow begins with the creation of a cost surface based on habitat suitability and the reduction of the observation data to the resolution of the cost surface. These inputs were then used to trace least-cost paths in annual time steps, connecting each new observation record to the nearest previously known record. Observations were then grouped into populations by excluding high-cost paths and examining the connectivity of the remaining paths. Finally, expansion rates were calculated for each population.

### Study species

**Fig. 2:**
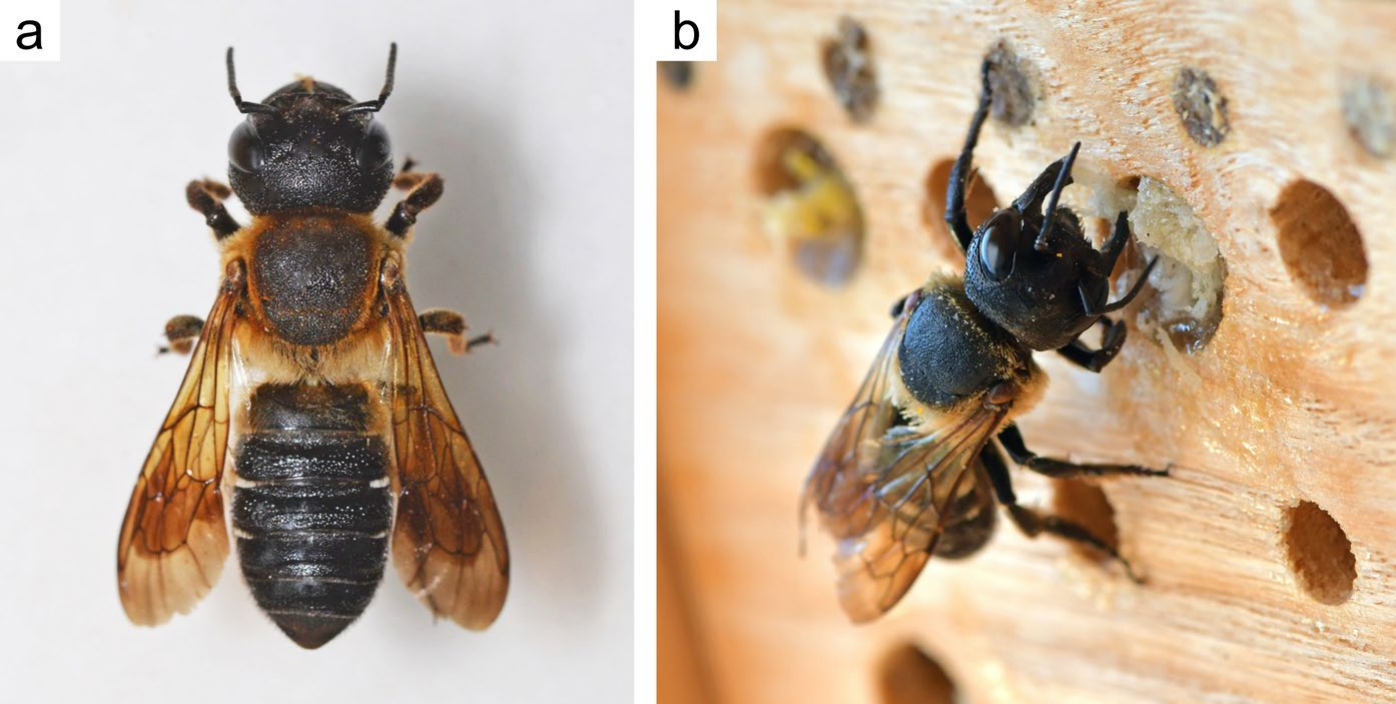
**a** Dorsal view of a female Sculptured Resin Bee ( © Philipp Heller, CC BY 4.0). **b** The species readily accepts artificial nesting aids. The picture displays a female preparing tree resin with its mouth parts to seal the nest entrance

The Sculptured Resin Bee (*Megachile sculpturalis* Smith, 1853) is a non-managed solitary bee species. Its native range is East Asia. Females are considerably larger than males and reach a body length of 22 to 27 mm (Parys et al. 2015). The species can be identified without specialised taxonomic knowledge due to its distinct size, slender body shape, dark colouration, and orange hair on its thorax (Lanner et al. 2020) (Fig. 2a). It builds nests in existing cavities in deadwood, and readily accepts artificial nesting aids (Fig. 2b). Both its appearance and nesting preference facilitate monitoring through Citizen Science projects (e.g. *BeeRadar.info* or *srbee.bio.bg.ac.rs/english*) and data collection via public sources, as for example Social Media and naturalist platforms. The bee is univoltine, producing a single generation per year. The first established population outside its native range was found in North Carolina, USA, in 1994 (Mangum and Brooks 1997). In Europe, it was initially observed in Allauch, France, in 2008 (Vereecken and Barbier 2009), and has since expanded the invaded area, now extending from the Pyrenees to the Caucasus. The development of the individuals in wood suggests that it may have reached Europe via timber trade (Quaranta et al. 2014), probably by multiple jump dispersals over long distances (Lanner et al. 2020, 2021).

### Study area

**Fig. 3:**
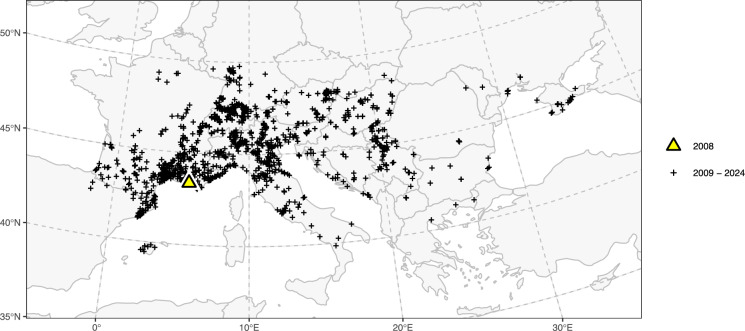
Spatial distribution of the observation records for the Sculptured Resin Bee in Europe. The data covers the period from the first observation in 2008 (yellow triangle) until 2024 (black crosses). The map extent corresponds to the study area

The study area encompasses the spatial extent of the observation records for the Sculptured Resin Bee, buffered by 400 km to provide sufficient space for the least-cost modelling algorithm, covering a large part of Europe (Fig. 3). To maintain a compact study area extent, two observations from the North and South Caucasus regions (in Mineralnye Vody, Russia, and Adjara, Georgia), published in 2024 on the Citizen Science platform *iNaturalist.org*, were excluded from the analysis due to their substantial distance from the nearest other observation (595 km and 631 km, respectively). The resulting study area stretches from the westernmost observation in the Atlantic Pyrenees, Spain, to the easternmost on the Crimean Peninsula, Ukraine, and from the southernmost observation in Calabria, Italy, to the northernmost in Lower Franconia, Germany.

### Data sources and preparation

Observation records for the Sculptured Resin Bee in Europe (Fig. 3) were provided by *BeeRadar.info* (Lanner et al. 2020), comprising 2245 partly unpublished records for the period from 2008 to December 2024. Most records (n = 865) were retrieved from Citizen Science platforms and Social Media sources (*beewatching.it, cardobs.mnhn.fr, entomologiitaliani.net, facebook.com, faune-france.org, flickr.com, inaturalist.org, inpn.mnhn.fr, insecte.org, instagram.com, izeltlabuak.hu, naturamediterraneo.com, naturbeobachtung.at, naturgucker.de, observation.org, silene.eu, spipoll.org*), followed by Citizen Science projects (n = 706) (*BeeRadar.info*, *srbee.bio.bg.ac.rs/english*). The remaining records were obtained from scientific literature (n = 629) (Vereecken and Barbier 2009; Amiet 2012; Gihr and Westrich 2013; Quaranta et al. 2014; Kovács 2015; Westrich et al. 2015; Fortel et al. 2016; Zandigiacomo and Grion 2017; Aguado et al. 2018; Gogala and Zadravec 2018; Grossi et al. 2018; Le Féon et al. 2018; Le Féon and Geslin 2018; Ortiz-Sánchez et al. 2018; Rickenbach and Sprecher 2018; Guariento et al. 2019; Ivanov and Fateryga 2019; Poggi et al. 2020; Polidori and Sánchez-Fernández 2020; Ruzzier et al. 2020; Westrich 2020; Lanner et al. 2020; Bila Dubaić et al. 2022a, 2022b; Gradinarov et al. 2023) and personal communication with scientific professionals (n = 45) (Giovanni, Celia; Guariento, Elia; Diaz Calafat, Joan; Bartolotti, Laura; Wanzenböck, Silvia; Dötterl, Stefan; Kovács, Tibor; Westrich, Paul). The data were projected from the original geographic coordinates (EPSG:4326) to the projection of the cost surface. For least-cost modelling, the observation records were reduced to the cost surface resolution, retaining one record per cell with the earliest observation year. This is required to avoid duplication of paths and zero-length paths, as the path-finding algorithm operates at the resolution of the cost surface, mapping input points to the nodes of the landscape graph.

**Fig. 4:**
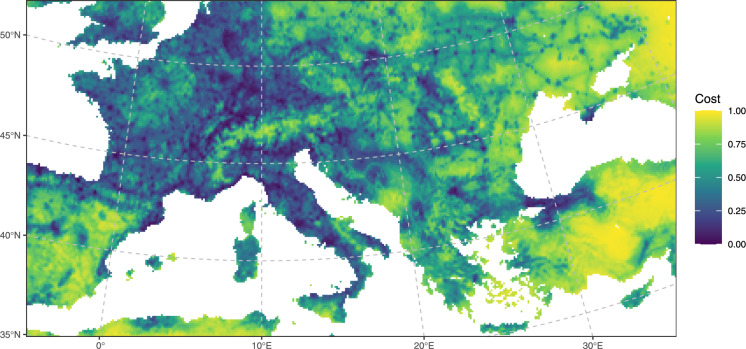
The cost surface used for least-cost modelling. It was derived from the mean prediction of various species distribution models for the Sculptured Resin Bee (Lanner et al. 2022) by reprojecting to an Equidistant Conic projection (ESRI:102031), clipping to the study area, and inverting and rescaling the value range

The cost surface was derived from an existing environmental suitability map for the Sculptured Resin Bee (Lanner et al. 2022), which represents a consensus of various species distribution models and was built using multiple predictor variables including bioclimatic factors (temperature and precipitation variables), vegetational features (tree coverage), and anthropogenic factors (distance to infrastructure and population density). We assumed that areas of higher environmental suitability would facilitate dispersal (low cost), while areas of lower suitability would impede dispersal (high cost). The original map was provided as a GeoTIFF file in the global equal-area Mollweide projection (ESRI:54009) and a spatial resolution of 10 km, with the ocean surface encoded as NoData. Biogeographical barriers on land such as the Alps were not specifically encoded but naturally appear as areas of low suitability in the original map. To reduce spatial distortion, the original raster was reprojected to an Equidistant Conic projection optimised for the spatial extent of Europe (ESRI:102031) using 4 × 4 cubic resampling. After clipping the file to the study area, the value range of [33.04,938.572] was inverted to model high suitability as low cost, and scaled to [0,1] (Fig. 4). These steps were executed in QGIS 3.34.5-Prizren.

### Least-cost modelling

The original least-cost modelling approach, established by the software *Generalized Computer-Aided Route Selection (GCARS)* (Turner and Miles 1971), is widely adopted due to its simplicity and integration into various software solutions (Driezen et al. 2007). It combines the following three methods: (1) modelling a landscape as a cost surface raster, where “cost” in this context reflects the difficulty of spread; (2) converting the cost surface to a graph based on a cell neighbourhood definition, allowing for 4-way (orthogonal), 8-way (orthogonal and diagonal), or 16-way (including non-adjacent cells via “Knight’s move”) movement from cell to cell; and (3) applying a path-finding algorithm to the landscape graph to determine the path of least cost between two specified nodes.

Least-cost paths were calculated with the *Graph* module of the Python package *scikit-image* (Walt van der et al. 2014), which supports graph-based least-cost modelling using an 8-way neighbourhood definition. The *MCP_Geometric* class was used to account for the geometric reality of movement across the landscape. Unlike the standard *MCP* class, which simply sums costs along a path, *MCP_Geometric* accumulates distance-weighted costs by accounting for the difference in distance between orthogonal and diagonal moves. The class is instantiated with an n-dimensional array; therefore, the cost surface and observation data were converted to a two-dimensional array and array coordinates, respectively, using the Python package *Rasterio* (Gillies et al. 2013). In this process, NoData values in the cost array were overwritten with infinite values, which are ignored by the path-finding algorithm. This effectively turns the ocean surface into an impassable barrier.

In our sequential approach to least-cost modelling, the algorithm was implemented in annual time steps to match the species’ univoltine life cycle. For each time step, costs were accumulated for connecting previously known observation records with new ones, followed by iteratively extracting the least-cost path for each new observation record. In this process, least-cost paths are created sequentially until least-cost paths for all observation records have been traced back, except for isolated observation records surrounded by a barrier. The result can be thought of as one large dispersal network, which contains the unfiltered entirety of least-cost paths, including paths with extremely high accumulated cost.

### Population delineation

To delineate populations, a four-step process was employed. First, least-cost paths that exceeded an accumulated cost threshold were removed from the result set. This isolated populations by dispersal cost. Second, unique population identifiers were assigned to the remaining paths based on their connectivity, defined by shared path endpoints. Third, the observation records were assigned to a population based on any path starting or ending in the same cost surface cell. Observation records isolated by barriers or by the removal of high-cost paths were thus not assigned to a population. These steps were executed using the Python packages *GeoPandas* (Jordahl et al. 2024), *NumPy* (Harris et al. 2020), *Pandas* (The pandas development team 2024), and *Shapely* (Gillies et al. 2024). In a fourth and last step, the spatio-temporal coverage of the populations with observation data was assessed by calculating the median observation count per year for each population. Populations with a median above 10 were classified as *robust*, indicating high confidence in their existence. In contrast, populations with a median below this threshold were classified as *sparse*, suggesting weaker evidence for their presence. The data coverage of sparse populations was regarded insufficient to adequately delineate populations and compute expansion rates and thus were ignored in the interpretation of results.

### Expansion rates

To determine the expansion rates, a distance regression approach was adopted, which regresses the cumulative Euclidean distance of the advancing invasion front to the first observation against time (Liebhold et al. 1992; Hui and Richardson 2017). The procedure was applied to each population and comprised the following three steps: (1) Distance calculation: The first observation record ${p}_{0}$ serves as the reference point. For each record ${p}_{i}$, calculate the Euclidean distance to ${p}_{0}$ using the *distance* function of the Python package *GeoPandas* (Jordahl et al. 2024). (2) Cumulative maximum distance: Using the distances, calculate the cumulative maximum for each year using the *cummax* function of the Python package *Pandas* (The pandas development team 2024). (3) Linear regression: Perform a linear regression of the cumulative maximum distance against year using an ordinary least squares model, based on the *OLS* class of the Python package *statsmodels* (Seabold and Perktold 2010). The slope of the regression line represents the expansion rate, which is divided by 1000 to convert the unit from meters per year (m/year) to kilometers per year (km/year).

To enable a better understanding of the temporal dynamics of the two populations with the longest temporal coverage, the regression was also conducted for three distinct phases. Breakpoints were determined manually based on abrupt changes in the expansion rate, as the limited number of data points (one per year), inherent to the distance regression method, precluded the use of statistically based methods for breakpoint detection. Independent models were fitted to each phase.

### Sensitivity analysis

Two parameters were found to be potentially critical in the presented approach, and thus were further tested in a sensitivity analysis to evaluate their impact on the average expansion rate across robust populations. First the *accumulated cost threshold*, which represents the maximum cost-distance that a bee can cover in its yearly dispersal, and second the *robust population definition*, expressed as the lower threshold of median observations per year. The sensitivity test was run in 6′792 steps of 0.01 across the full range of accumulated cost of least-cost paths (0 to 67.91) and for varying robust population definitions from 5 to 15 median observations per year.

## Results

We present our findings following our analytical reasoning rather than the chronological order of our methodological steps. We begin with results from the sensitivity analysis, as this provided crucial insights for determining optimal parameter values for population delineation. Following this foundation, we present the delineated populations and their characteristics and then proceed with analysing expansion rates for *robust populations*, i.e. those exceeding a median of 10 observations per year.

### Model sensitivity and identification of parameter values

**Fig. 5:**
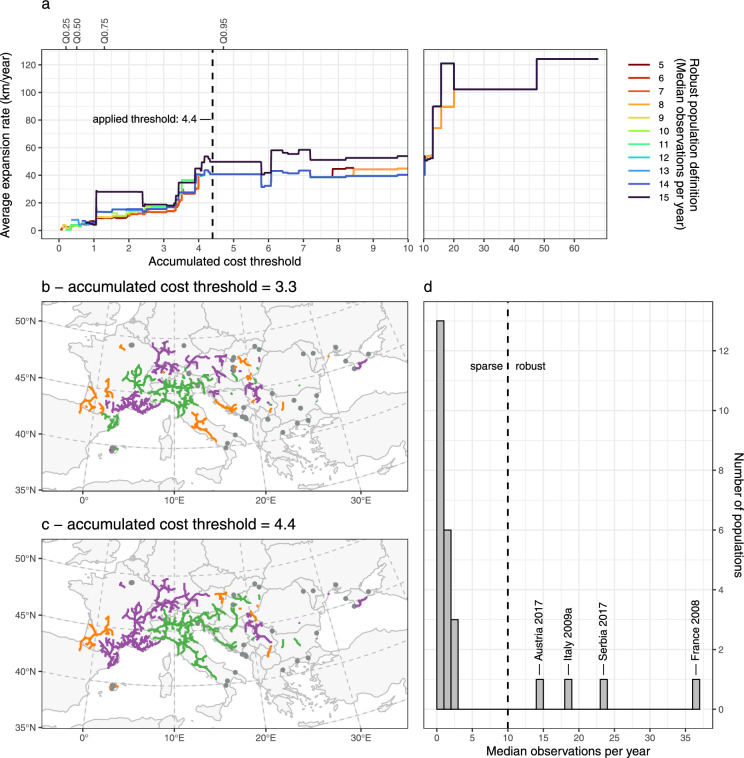
Sensitivity analysis of two critical parameters affecting average expansion rates across robust populations that were delineated using a least-cost modelling based approach. **a** Effect of accumulated cost threshold (maximum yearly dispersal capacity) and robust population definition (minimum observation data coverage) on expansion rates. Different lines represent varying robust population definitions (5–15 median observations per year). The vertical dashed line indicates the selected accumulated cost threshold of 4.4 (94.7% quantile), which falls at the beginning of a stable plateau (4.4-13) where expansion rates remain consistent. **b** Artificial population fragmentation at a low accumulated cost threshold of 3.3. **c** Populations delineated with an adequate accumulated cost threshold of 4.4. At this threshold, both artificial fragmentation of populations (thresholds < 4.4) and unrealistic merging of distant populations (thresholds > 13) are avoided. **d** Distribution of observation data coverage (median observations per year) at an accumulated cost threshold of 4.4. Most populations exhibit a low data coverage below four median observations per year, and four populations reach more than 14 median observations per year. The selected robust population definition of 10 median observations per year falls within an insensitive range (4-14)

The results of the sensitivity analysis revealed a nonlinear relation of the accumulated cost threshold parameter on the resulting average expansion rate of robust populations (Fig. 5a). At low accumulated cost thresholds (0 to 3.3), expansion rates increased steadily due to the inclusion of longer paths, followed by a phase between threshold values of 3.3 to 4.3 where a high number of small populations was merged into larger populations (Fig. 5b, c) leading to a steep increase of expansion rates. This nonlinear impact of increasing accumulated cost thresholds on expansion rates was expected for thresholds that are below the dispersal capacity of the bee. It suggests that biological populations are artificially fragmented and thus yield lower expansion rates due to constrained maximum distances from first observations. Between 4.3 and 13, expansion rate estimates remained remarkably stable despite increasing thresholds, indicating an insensitive range. Values above 13 included only seven extreme high-cost paths (out of 886) that merged populations across unrealistic distances, creating biologically implausible super-populations spanning a large part of the study area.

The second parameter, the robust population definition, expressed as the lower threshold of median observations per year, only had marginal influences on the resulting average expansion rate in a large insensitive range between 5 and 14 median observations per year (Fig. 5a, d). Only a high threshold of 15 median observations per year eliminated the robust population with the lowest expansion rate *(Austria 2017)*, driving the average expansion rate higher (Fig. 5a, d).

Considering these patterns, an accumulated cost threshold of 4.4 was identified to be most adequate for the delineation of populations, as it falls at the beginning of the insensitive accumulated cost threshold range. This threshold represents the 94.7% quantile in the distribution of the accumulated cost of least-cost paths. To define robust populations, a threshold value of 10 median observations per year was applied, as this value lies in the middle of the insensitive range.

### Population delineation

**Fig. 6:**
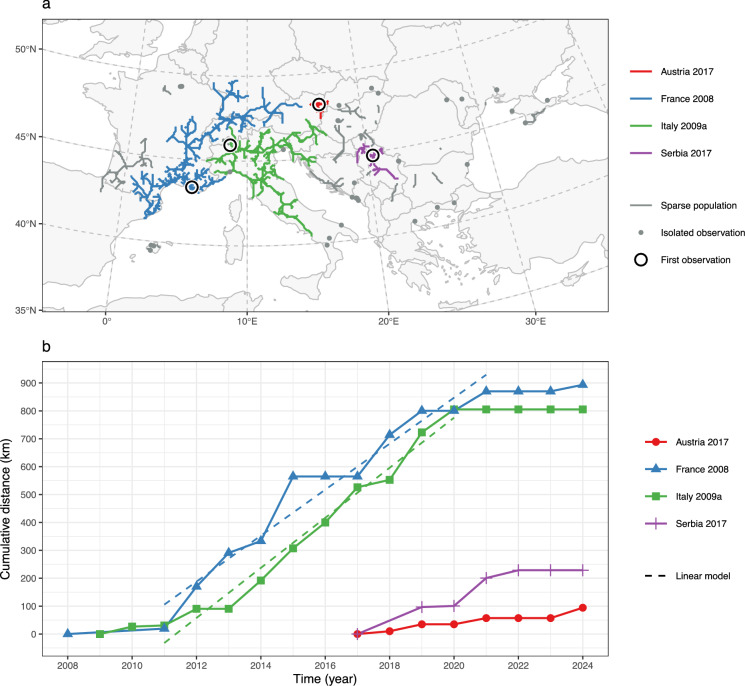
**a** Population assignment of the least-cost paths, which connect the observation records. Robust populations are symbolised in colour. Sparse populations and unassigned isolated observations are symbolised in grey. **b** Diagram showing the increase in cumulative distance of observations to the first observation over time for robust populations. The two dashed lines represent the linear models fitted to the respective expansion phase, starting in 2011 and ending in 2021 (population France 2008) and 2020 (population Italy 2009a), respectively

For the analysed period from 2008 to 2024, 886 least-cost paths were generated, of which 839 remained in the result set after application of the accumulated cost threshold (4.4 / 94.7% quantile). By subsequently assigning population identifiers to connected least-cost paths and nearby observation records, 26 populations were delineated (Table 1, Fig. 6a), with 37 observation records remaining unassigned due to isolation by the ocean barrier or by the removal of high-cost paths. Online Resource 1 shows new observations and associated least-cost paths for each time step of the sequential least-cost modelling process.

**Table 1:** All populations delineated using a least-cost modelling based approach alongside expansion rates and ${\text{R}}^{2}$ value of the linear regression model used to calculate the expansion rates

	Observation records					Expansion	
	First (country)	First (year)	Latest (year)	Count (n)	Median (n/year)	Rate (km/year)	Model strength (${R}^{2}$)
France 2008*	France	2008	2024	1,000	37.0	52.4	0.85
Italy 2009a*	Italy	2009	2024	542	18.5	58.6	0.78
Austria 2017*	Austria	2017	2024	202	14.5	13.3	0.83
Serbia 2017*	Serbia	2017	2024	238	24.0	38.3	0.68
Italy 2009b	Italy	2009	2021	14	1.0	6.4	0.79
France 2015	France	2015	2024	37	2.5	15.4	0.66
Hungary 2015	Hungary	2015	2024	28	2.5	9.5	0.67
France 2016	France	2016	2024	3	0.0	10.2	0.86
Croatia 2018	Croatia	2018	2024	15	2.0	23.4	0.78
Hungary 2018a	Hungary	2018	2024	19	3.0	21.4	0.82
Hungary 2018b	Hungary	2018	2024	5	0.0	6.1	0.87
Serbia 2018	Serbia	2018	2024	19	2.0	6.0	0.41
Ukraine 2018	Ukraine	2018	2024	18	1.0	16.2	0.71
Hungary 2018c	Hungary	2018	2021	3	0.5	9.5	1.00
Hungary 2019a	Hungary	2019	2024	19	1.5	19.3	0.29
Romania 2019	Romania	2019	2024	5	1.0	1.6	0.74
Hungary 2019b	Hungary	2019	2021	8	2.0	9.3	0.88
Spain 2020a	Spain	2020	2024	2	0.0	3.7	1.00
Spain 2020b	Spain	2020	2023	2	0.5	9.6	1.00
Serbia 2020	Serbia	2020	2022	8	2.0	16.6	0.74
Hungary 2021	Hungary	2021	2024	2	0.5	14.9	1.00
Ukraine 2021	Ukraine	2021	2024	8	1.0	3.7	0.32
Serbia 2021	Serbia	2021	2023	4	1.0	62.4	0.99
Bulgaria 2021	Bulgaria	2021	2022	3	1.5	103.4	1.00
Bulgaria 2022	Bulgaria	2022	2023	2	1.0	91.5	1.00
Bulgaria 2023	Bulgaria	2023	2024	2	1.0	10.6	1.00

Robust populations are displayed at the top and marked with an asterisk (*). The table is sorted by first observation year (ascending) and latest observation year (descending)

Four delineated populations were classified as robust populations, with a median observation count per year ranging from 14.5 to 37: *Austria 2017*, *France 2008*, *Italy 2009a*, and *Serbia 2017*. The remaining 22 populations were classified as sparse populations and have less than four median observations per year (Fig. 5d).

### Expansion rates

Expansion rates in robust populations range from 13.3 km/year to 58.6 km/year, with an average rate of 40.6 km/year. The highest expansion rate of 58.6 km/year is observed in the population *Italy 2009a* originating in Northern Italy, while the lowest is 13.3 km/year in the population *Austria 2017* originating in Vienna, Austria (Table 1). This population covers the smallest spatial extent with a concentration of observation records in Vienna. The two populations with the largest temporal coverage, populations *France 2008* (16 years) and *Italy 2009a* (15 years), exhibit a sigmoidal increase in cumulative distance from the first observation location. Their observation period was divided into three phases, with a lag phase until 2011 and an expansion phase until 2021 (*France 2008*) and 2020 (*Italy 2009a*), respectively. In their expansion phase, the two populations reached an expansion rate of 82.4 km/year (*France 2008*) and 89.7 km/year (*Italy 2009a*) (Table 2). In 2022 and 2023, no increase in cumulative distance was observed in any of the robust populations. In 2024, however, a significant increase by a factor of 1.65 was recorded for the *Austria 2017* population.

**Table 2:** For the two populations with the largest temporal coverage and sigmoidal expansion pattern, the time span was split into three expansion phases and expansion rates were calculated separately for each phase

Population	Phase	From	Until	Expansion rate (km/year)	Expansion rate model strength (R2)
France 2008	Lag phase	2008	2011	6.5	1.00
	Expansion phase	2011	2021	82.4	0.95
	Final phase	2021	2024	7.0	0.60
Italy 2009a	Lag phase	2009	2011	15.4	0.85
	Expansion phase	2011	2020	89.7	0.98
	Final phase	2020	2024	0.0	N/A

## Discussion

This research inferred the population structure of the multiply introduced Sculptured Resin Bee from observation data using a sequential least-cost modelling based approach. The main findings include (1) a spatio-temporal delineation of distinct populations, and (2) their respective expansion rates. As expected, the two populations with the largest temporal coverage show a sigmoidal expansion pattern, including an initial lag phase, a rapid expansion phase, and a slow final phase. In the discussion of expansion rates, we focus on the expansion phase, as this phase represents the period of greatest spatial spread following establishment in a new geographic region and is recommended to be used in invasive species management (Sandvik 2020).

### Least-cost modelling

As a prerequisite for least-cost modelling, we created a cost surface from an existing environmental suitability map (Lanner et al. 2022). Notably, our modelled populations align well with existing genetic evidence (Lanner et al. 2021; Lanner et al. unpublished data), as discussed in the next chapter, suggesting the validity of applying this approach to the Sculptured Resin Bee. While habitat suitability and dispersal permeability are conceptually distinct (Adriaensen et al. 2003; Zeller et al. 2012), Diniz et al. (2020) observe that for certain species, habitat suitability can serve as an appropriate proxy for dispersal cost, though habitat selection patterns may vary depending on movement purpose. Balbi et al. (2021) empirically validated this approach, demonstrating that least-cost paths based on habitat suitability predicted functional connectivity for moths and birds in urban environments.

The original least-cost modelling approach as implemented in this study has two widely recognized limitations. First, the movement in a graph creates path distortions due to the constrained directions of movement (Goodchild 1977), affecting path direction and length. Although this has been addressed by alternative implementations, which avoid the graph conversion (Douglas 1994; Tomlin 2010), Adriaensen et al. (2003) pointed out that in ecological applications this algorithmic limitation is likely far less impactful than uncertainties in the estimation of landscape model parameters. Furthermore, we focused on broader population-level patterns rather than precise dispersal pathways. Neither the geometry, nor the length or accumulated cost of paths were processed further. The second limitation is the deterministic outcome of an optimal path that may not realistically represent animal movement (Etherington 2016). While probabilistic modifications of least-cost modelling exist (Pinto and Keitt 2009; Saerens et al. 2009; Etten 2017; Lewis 2021; Van Moorter et al. 2023), Etherington (2016) hypothesizes that original least-cost modelling might be particularly well-suited for studies where an optimistic view of connectivity is required, such as for invasive species management. Also, we used least-cost paths to model a spatio-temporal relationship between observation records, rather than actual dispersal pathways of individual bees or gene flow. Therefore, we placed greater emphasis on the simplicity and broad applicability of our approach than on algorithmic optimisation.

### Population delineation

We identified four robust populations in France, Italy, Serbia, and Austria. The delineation of these populations closely aligns with the biogeographical barrier of the Alps, even though the Alps were not encoded as impassable barriers, unlike the ocean surface. While a single least-cost path does unexpectedly cross the Alps in a south-north direction (Fig. 6a), this is likely an artifact of spatial scale. The coarse resolution of the habitat suitability map (10 km) averages the underlying environmental conditions in this highly structured area, potentially obscuring fine-scale barriers that would naturally impede dispersal.

**Fig. 7:**
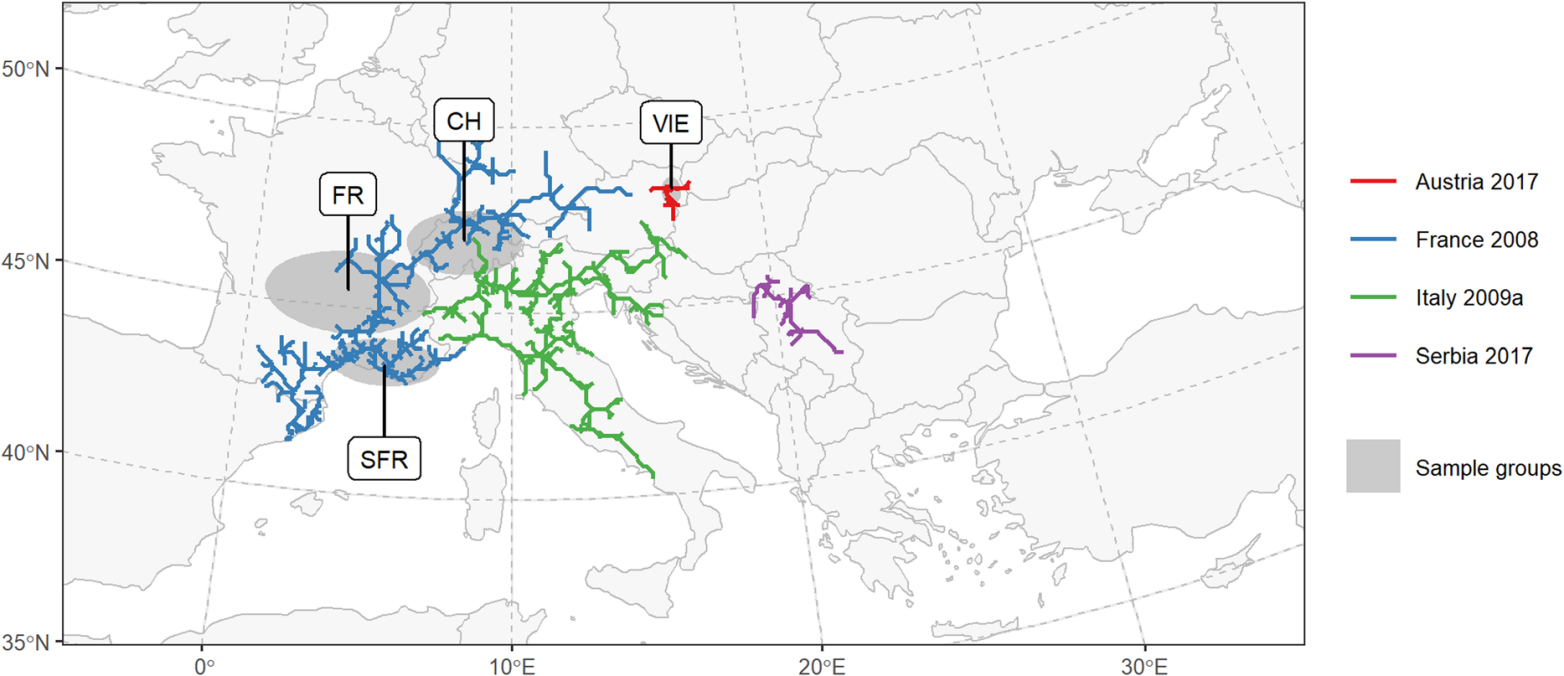
The populations delineated using a least-cost modelling based approach align with genetic groups. Lanner et al. (2021) found strong genetic evidence for at least two independent introductions, with one genetic group formed by the sample groups SFR, FR and CH, and another genetic group formed by the sample group VIE. The sample groups shown here are an approximation of the actual sample groups (cf. Lanner et al. 2021)

Our modelled populations show strong concordance with genetic evidence from Lanner et al. (2021), who identified at least two genetic groups: one consisting of samples from Vienna, Austria (sample group *VIE*), and another consisting of samples from France and Switzerland (sample groups *SFR*, *FR*, *CH*) (Fig. 7). Using purely spatio-temporal methods, our approach reconstructed these same groups, delineating the *Austria 2017* population in the greater area of Vienna and the *France 2008* population spreading eastward along the north side of the Alps. While statistical validation is constrained by the limited number of georeferenced genetic samples provided by the aforementioned study (26 samples from 12 sampling sites out of 72 total samples), all georeferenced samples (100%) were correctly grouped by our delineation approach.

Based on our results and the robust data coverage, especially in Italy, we hypothesise that the *Italy 2009a* and *Serbia 2017* populations are also the result of independent introduction events. This hypothesis is supported by preliminary results from an extended genetic sampling effort that also supports the modelled populations *Italy 2009a* and *Serbia 2017* (Lanner et al., unpublished data).

### Expansion rates

The two populations with the largest temporal coverage showed high expansion rates in their expansion phases, reaching 82.4 km/year (*France 2008*) and 89.7 km/year (*Italy 2009a*). These rates are surprisingly high given the species’ known homing range of 10 km, as determined in translocation experiments (pers. comm., Lanner 2024). While flight capabilities of this bee species have not been quantified in experiments, expansion rates of 80 km/year and more challenge biological explanations. We examine four factors that may contribute to the observed high expansion rates.

1. Active dispersal flights: Dispersal flights differ fundamentally from homing flights, often covering large distances over several days (Chapman et al. 2011). For example, male orchid bees have been recorded dispersing up to 95 km in mark-recapture experiments (Pokorny et al. 2015). Dispersal flight behaviour likely contributes significantly to the rapid expansion identified in this study.
2. Wind-assisted dispersal: Active dispersal flights may be further aided by wind currents. The eastward-directed expansion pattern, particularly evident in the population France 2008, aligns with the prevailing westerly winds in the study area. However, while wind assistance is well-documented for migratory insect species (Chapman et al. 2011; Reynolds et al. 2024), it has not been reported for bees. Further research is needed to shed light on the potential role of wind in bee dispersal.
3. Human-mediated jump dispersal: Human activities are a recognized driver of invasive species expansion, transporting invasive organisms over long distances (Trakhtenbrot et al. 2005; Wilson et al. 2009; Hui and Richardson 2017; Gippet et al. 2019). However, the random distances characteristic of human-mediated translocations conflict with the steady increase in cumulative distance with a strong model fit for expansion phases (${R}^{2}$ = 0.95 and ${R}^{2}$ = 0.98, respectively). Furthermore, as we delineate populations based on an accumulated cost threshold, we effectively limit the distance covered within a year. This approach does not only delineate populations resulting from independent introductions, but also subpopulations resulting from long-distance jump dispersal.
4. Methodological issues: Errors in population delineation could theoretically affect expansion rate estimates. Overpartitioning a single population could result in lower rates for the reduced populations, while underpartitioning multiple populations could inflate expansion rates. However, as discussed earlier, our population delineation approach has been validated in parts of the study area. Nonetheless, observational biases may impact the expansion rate estimates. For example, only genetic analyses could determine whether the population France 2015 in southwestern France originated from an independent introduction event, from human-mediated jump dispersal originating in population France 2008, or if the population was incorrectly delineated due to observational biases.

### Potential observational biases

Certain limitations should be acknowledged, particularly observational biases affecting the underlying data of the study. These biases arise from (1) targeted monitoring efforts and (2) the spatio-temporally uneven distribution of Citizen Science contributions.

1. Targeted monitoring efforts: Later-detected populations (Austria 2017 and Serbia 2017) were subject to targeted monitoring efforts following initial observations (Bila Dubaić et al. 2022b). These efforts increased observation data density but were limited to a constrained spatial area. We anticipate that the Austria 2017 and Serbia 2017 populations are observed at an early invasion stage and that an accelerated expansion phase will be detectable in the near future. In contrast, populations detected earlier (France 2008 and Italy 2009a) lacked targeted large-scale monitoring efforts, building primarily on opportunistic observations from the public (Le Féon et al. 2018; Flaminio et al. 2021).
2. Spatio-temporally uneven Citizen Science contributions: When the first populations (France 2008, Italy 2009a) were detected in 2008 and 2009, respectively, public Citizen Science platforms like iNaturalist.org (founded in 2008) were less established than they are today. Further, in Southeast Europe, Citizen Science remains of limited prevalence (Golubović et al. 2019; Bila Dubaić et al. 2022a). Language barriers also contribute to uneven data contributions. For example, Romania appears as a largely blank spot on the observation data map, probably partly because the contributing Serbian Citizen Science project (srbee.bio.bg.ac.rs/english) faces greater challenges in reaching an audience in Romania compared to the Slavic-speaking regions. Additionally, the species has spread beyond the primary focus area of the contributing Citizen Science projects BeeRadar.info (Switzerland, Liechtenstein, Austria) and srbee.bio.bg.ac.rs/english (Serbia). As a result, we expect reduced observation rates during the early study period, in the geographic region of Southeast Europe, and outside the project focus areas.

Another limitation relates to observation data density, which is critical for robust population delineation. In regions with low-density observation data, such as parts of Southeast Europe, the process may produce unrealistically fragmented populations with very limited data coverage, or leave isolated observations unconnected to any population. While this limits the ability to achieve the desired results, the fragmented pattern highlights potential observation gaps, providing valuable insights for guiding future monitoring projects.

## Conclusion

This multidisciplinary study demonstrates the utility of a data-driven and spatio-temporal framework for analysing the spread dynamics of invasive species. By employing a sequential least-cost modelling based approach, we successfully inferred the population structure of the invasive Sculptured Resin Bee in Europe, suggesting four populations likely resulting from multiple introductions. The delineation of these populations was validated against existing genetic evidence in part of the study area, and their expansion patterns exhibit remarkable consistency. In addition to this comparison with genetic evidence, two additional criteria supported the validity of our results: (1) the identified expansion rates during expansion phases between 80 and 90 km per year appear high, but were shown to be biologically plausible, and (2) the results proved to be robust against systematic variation of key parameters. These findings underscore the potential of our approach to support management of invasive species by informing targeted monitoring and genetic sampling efforts.

Future studies should aim to validate our novel population delineation approach more comprehensively by expanding genetic sampling of this bee species across the entire study area and by applying the approach to other invasive species. Additionally, extending this framework from retrospective to predictive modelling—for example, based on regressing accumulated costs against time rather than Euclidean distance—could enable projections of future spread, providing valuable support for proactive management strategies.

Finally, our findings raise important ecological questions about our study species, particularly regarding the observed and consistent high expansion rates exceeding 80 km/year during expansion phases. We hypothesise that multiple factors, such as a strong dispersal ability of the bee and favorable wind conditions assisting the dispersal, may have contributed to these rates. Future research could test for significant directional dependencies in the expansion patterns, while the directional information inherent in least-cost paths offers a promising avenue for such analyses.

## Supplementary Information

Below is the link to the electronic supplementary material.

Supplementary file 1.

## Data Availability

The Python code used to generate the results is freely available at https://github.com/thunwal/bioinvasionanalysis/releases/tag/v1.1.0. The data analysed and produced is available from the corresponding author upon reasonable request.
